# Heat Treatment of Hazelnut Allergens Monitored by Polyclonal Sera and Epitope Fingerprinting

**DOI:** 10.3390/foods13233932

**Published:** 2024-12-05

**Authors:** Karolin Kern, Suttinee Santa-Ardharnpreecha, Nicolas Delaroque, Sabine Dölle-Bierke, Regina Treudler, Eva Ehrentreich-Förster, Isabell Rothkopf, Margitta Worm, Michael Szardenings

**Affiliations:** 1 Department Diagnostics, Fraunhofer Institute for Cell Therapy and Immunology (IZI), Perlickstr. 1, 04103 Leipzig, Germany; karolin.kern@izi.fraunhofer.de (K.K.); nicolas.delaroque@izi.fraunhofer.de (N.D.); 2 Division of Allergy and Immunology, Department of Dermatology, Venerology and Allergy, Charité-Universitätsmedizin, 10117 Berlin, Germanymargitta.worm@charite.de (M.W.); 3 Institute of Allergology (IFA), Charité-Universitätsmedizin, 12203 Berlin, Germany; 4 Bioanalytics and Bioprocesses Branch, Fraunhofer Institute for Cell Therapy and Immunology (IZI), Am Mühlenberg 13, 14476 Potsdam-Golm, Germany; eva.ehrentreich@izi-bb.fraunhofer.de; 5 Fraunhofer Institute for Process Engineering and Packaging IVV, 85354 Freising, Germany; isabell.rothkopf@ivv.fraunhofer.de; 6 Epitopic GmbH, Deutscher Platz 5e, 04103 Leipzig, Germany

**Keywords:** epitope fingerprints, thermal processing, rabbit sera

## Abstract

Hazelnuts are frequently involved in IgE-mediated reactions and are the main cause of nut allergies in Europe. Most food products are processed before human consumption. Food processing can modify the structure, properties, and function of proteins, and as a result, the IgE-binding capacity of allergens can be affected. In this study, we aimed to investigate epitope changes caused by the roasting of hazelnuts using epitope fingerprinting. Rabbit sera were raised against hazelnut proteins, and their epitopes were characterized. Immunoassays using specific polyclonal antibodies from rabbits targeting the main allergens in hazelnuts revealed marked reductions in the levels of Cor a 1 (PR-10), Cor a 11 (7S globulin), and Cor a 14 (2S albumin). However, rabbit antibodies can recognize different epitopes. Using antibodies that are different and characterized could help establish reliable methods for estimating the effects of treatments on the allergenicity of foods. In this work, we provide the first practical application that could lead to sets of peptide epitopes to compare and standardize immune diagnostics, even for complex protein preparations.

## 1. Introduction

Tree nuts are popular worldwide. They are beneficial food sources because they are rich in unsaturated fats and have comparatively high levels of phenolics, phytosterols, tocopherols, minerals, and fiber [1]. This may explain why the consumption of both raw and processed tree nuts is increasing. In recent years, there has been an increase in tree nut consumption because of its favorable health effects [2]. For example, a diet rich in tree nuts has been shown to positively affect cardiovascular-risk biomarkers [3]. In Europe, tree nuts are among the predominant foods involved in allergic reactions to plants and may cause anaphylaxis [4]. The prevalence of nut allergy in Central and Northern Europe ranges from 1–2% [5], and hazelnuts in particular are one of the most common triggers of food-induced anaphylaxis in children and adults [6]. Most nut allergens are highly resistant to enzymatic degradation and denaturation. Several allergenic proteins of hazelnut have been registered in the WHO-IUS list of allergenic proteins: Cor a 1 (Bet v1 homolog), Cor a 2 (profilin), Cor a 8 (Lipid Transfer Protein LTP), Cor a 9 (11S legumin), Cor a 11 (7S vicilin), Cor a 14 (2S albumin), Cor a 12, Cor a 13, and Cor a 15 (oleosins) [7]. In general, hazelnut allergies can be of two forms: primary allergic sensitization to heat-stable hazelnut proteins or secondary food allergy due to immunological cross-reactivity between the structurally related Bet v 1 (a major birch pollen allergen) and members of the pathogenesis-related protein 10 (PR-10) family [8].

Most food products must be processed before human consumption to improve safety, functionality, and organoleptic properties. Unit operations involving the use of heat, such as pasteurization, sterilization, boiling, frying, roasting, and drying, are among the most common operations in food processing. Food processing can modify the structure, properties, and function of proteins, and as a result, the IgE-binding capacity of allergens can be affected. Some processing methods can effectively reduce the content of specific allergens but do not completely abolish them. As some processing treatments have been shown to decrease the allergenicity of certain foods, food processing may play an important role in the development of hypoallergenic foods and the induction of food tolerance [1,9]. Other processes can also increase the allergenicity of certain foods [9]. Roasting, defined as dry heat treatment, is one of the most important processes that provides the product with the required alterations to become value-added nuts. Hazelnut roasting times of 30–50 min at 142 °C are the industrial standard, commonly referred to as low-temperature–long-time (LTLT) processes. In contrast, modern food processing increasingly employs high-temperature–short-time (HTST) methods to preserve raw material quality, including nutritional properties. HTST roasting systems, such as infrared, fluidized bed, or accelerated drum roasting, achieve roasting times of approximately 10 min. This study contrasts LTLT and HTST roasting processes, ensuring sufficient differences in conditions to observe distinct effects [10,11].

These treatments can induce changes in the chemical properties of proteins or biochemical reactions inside food matrix components [12,13]. Numerous studies have been conducted on the modification of the allergenicity of tree nuts using different heat treatments [12,14,15]. Using oral provocation tests, the heat processing of hazelnuts has been shown to reduce allergenicity [16].

Rabbit serum is used to characterize food allergenicity [17,18,19]. Rabbit serum has been used, amongst others, for the investigation of changes in allergenicity after heat processing [17], cross-reactivity between almond and *Prunus mahaleb* [18], and cross-reactivity between animal IgG and human IgE antibodies [19]. These examples demonstrate that rabbit serum can probably replace human serum in investigations of food allergenicity; however, previous studies have not characterized immune responses at the epitope level. 

Sensitization against antigens is currently determined by IgE measurement of the antigenic protein, although it may be assessed much better at the level of individual allergy-relevant peptide epitopes. In addition, IgE-binding epitopes that are often less than 20 amino acids long are more suitable [20]. Several methods have been reported for mapping IgE-binding epitopes [21]. Arrays of overlapping peptides synthesized on a nitrocellulose membrane (SPOTmembrane) [22] can be used to determine sequential epitopes [23,24]. Overlapping peptides can also be used in an enzyme-linked immunosorbent assay (ELISA) format [25]. However, this approach is more effective and frequently used in microarray formats because many more peptides can be used with less serum [26,27,28]. However, the determination of conformational epitopes requires sophisticated techniques such as peptide derivatives [29], nuclear magnetic resonance, mass spectrometry, mutant generation, and in silico analysis [30,31]. Phage displays can be used for linear or conformational epitope mapping [32,33,34].

In this study, we aimed to investigate epitope changes caused by the roasting of hazelnuts using epitope fingerprinting. Rabbit sera were raised against hazelnut proteins and their epitopes were characterized [33]. Epitopes similar to those previously identified in serum samples from hazelnut-sensitized patients were also identified. These peptide epitopes were confirmed using peptide microarrays with rabbit anti-hazelnut polyclonal antisera. Most epitope peptides were bound to IgG from rabbit sera. Western blot analysis was performed to investigate the cross-reactivity with other proteins from legumes and tree nuts.

## 2. Materials and Methods

### 2.1. Hazelnut 

Forty-eight raw and undamaged hazelnuts with a diameter of 11–13 mm were roasted in a convection oven with a Pyrotherm EEBP 6400.8 MX from Küppersbusch, Gelsenkirchen, Germany, under the following four conditions: 142 °C for 30 min, 142 °C for 50 min, 200 °C for 8 min, and 200 °C for 10 min.

To ensure that the roasts were comparable, the nuts were roasted at the same roasting temperature in the same oven and height. The roasted hazelnuts were ground using a food processor (Moulinette DPA1X; Tefal, 73312 Geislingen/Steige, Germany) to obtain a fine powder.

Roasting conditions were selected as two typical ways to achieve the same roasting color: low-temperature–long-time vs. high-temperature–short-time. Shorter times at low temperatures and longer times at high temperatures were assumed to cause less or more damage to the nuts, respectively.

### 2.2. Animal Sera

Immunization of rabbits and preparation of sera were carried out by Seramun Diagnostica GmbH (Spreenhagener Str. 1, 15754 Heidesee, Germany; permit 2347-15-2023-20-E, LAVG Brandenburg). Rabbits were immunized with de-oiled hazelnut flour and complete Freund’s adjuvant. A boost immunization with incomplete Freund’s adjuvant was performed on day 21. Rabbits were bled after 28 days, and sera were used for phage display and array experiments.

### 2.3. Patient Sera

The human blood samples from patients allergic to tree nuts used in this study were provided by Charité-Universitätsmedizin Berlin (Department of Dermatology, Venerology, and Allergy) and Universitätsklinikum Leipzig (Department of Dermatology, Venerology, and Allergy). The use of samples for this study was authorized by the responsible ethics committee (Ethics Committee of the Medical Faculty of Leipzig University (AZ202/16-ek) and Charité’s Ethics Committee (EA01/022/20). Donors were informed that the blood samples would only be used for research purposes. Each donor signed a statement regarding the informed consent.

### 2.4. Peptide Phage Display Experiments

A special naïve peptide phage display was used in the selection experiments [32], and only two selection rounds were performed, as previously described [32,33]. Briefly, a selection from the ENTE-1 peptide phage display library was performed using Dynabeads Protein A (Thermo Fisher Scientific, Waltham, MA, USA). Two rounds of selections were performed. Pooled DNA of the recovered phagemids from the first and second rounds of selection was subjected to next-generation sequencing (NGS) on an Illumina MiSeq, as previously described. The back and forward runs were combined using PEAR (Version 0.9.6 [35])and processed using Trimmomatic (Version 0.33 [36])). Finally, datasets from each sequencing run were cured of sequencing errors and other artifacts, as described in the latest version of LibDB software (Version 1.34, Epitopic GmbH, Leipzig, Germany). Any sequences deviating from the library codon structure were sorted using this procedure because they potentially contained additional reading errors from the sequencer.

#### Identification of Epitopes by Motifs

Sequences statistically enriched by binding to serum antibodies and sharing motifs resembling antigen sequences were selected from NGS datasets, as previously described [32,37]. Sequences with motifs matching the antigens were retrieved from sequence pools obtained after the first or second round of selection. The alignment of sequences identified longer stretches of similarity to the antigen as well as structural features such as Cys-constrained peptides in a few hundred sequences. Using a naïve library allows for the search for epitopes that match multiple antigens in the dataset of a single selection experiment.

### 2.5. IgG-Binding Measurements Using Peptide Microarrays

#### 2.5.1. Selection of Peptide Epitopes/Mimotopes for Spotting on Microarray Slides

Peptides were derived from either native hazelnut antigen sequences (epitopes) or mimotopes obtained from phage-displayed sequences in selection experiments. Selected peptides and their origins are listed in Supplementary Information.

#### 2.5.2. Peptide Microarrays

The peptides were purchased from Peptides & Elephants GmbH (Hennigsdorf, Germany). All peptides had a C-terminal ebes-ϵ-azido-Lys linker; therefore, they were printed and immobilized in triplicate on DBCO-coated glass slides using click chemistry, as previously described [32,37].

The slides contained two clusters of 287 peptide epitopes, spotted in a quadruple. We measured the binding of IgG to peptide epitopes.

Each slide was blocked for 1 h at 4 °C in array buffer (PBS containing 0.1% *v/v* Tween^®^ 20 and 1% *w*/*v* casein, pH 7.4). The slides were incubated for 1 h at RT with rabbit anti-hazelnut polyclonal antiserum diluted 1:3000 in array buffer, washed twice (PBS containing 0.1% *v*/*v* Tween^®^ 20, pH 7.4), and incubated for 1 h at RT in a solution of Cy5-labeled goat anti-mouse antibody diluted 1:5000 in array buffer (ThermoFisher Scientific, Waltham, MA, USA). Finally, the slides were washed twice, and fluorescence was measured in a microarray reader at 10 μm resolution using a laser at 532 nm with 25% power/PMT Gain 600 and 635 nm with 25% power/PMT Gain 600 (Genepix 4300; Molecular Devices, San Jose, CA, USA).

#### 2.5.3. Determination of Spot Intensities

This method has been described previously [32,37]. The first step is the detection of the a priori known grid (GAL file) in the image, as the grid may be rotated or shifted. Using the known position of each spot, we used a segmentation approach that combines a seeding threshold and masking threshold with geodesic dilation to distinguish between the foreground and background signals. In the last step of image analysis, we extracted information regarding the fluorescence intensity and shape of the segmentation. As a reliable and robust method to decide on a positive or negative spot result, we used the total count of all pixels in the spot after subtracting the background per pixel, summarizing all intensities in a spot segment, and subtracting the median of the block background for every pixel.

The raw total fluorescence signal intensities of the quadruples were used to calculate the upper and lower quartiles. The difference between the upper and lower quartiles was multiplied by 1.5. This number was added to the upper quartile and subtracted from that of the lower quartile. All raw fluorescence signal intensities that were not within the calculated range were excluded. The adjusted raw fluorescence signal intensity was calculated as a multiple of the background (water) signal.

### 2.6. SDS-PAGE and Western Blot

#### 2.6.1. Sample Extraction and SDS-PAGE

The extraction protocol was performed as described by Dooper et al. [38], and ground hazelnut (50 mg) was suspended in 5 mL 0.05 M Tris-HCl buffer (pH 7.5) by vortexing for 5 min at room temperature Then, it was left at room temperature overnight, yielding three layers the next day: the fat, water, and ground nut layers. Only water layers were collected. The remaining extract was discarded.

Then, 21 μL of the sample was mixed with 7 μL SDS sample buffer. The mixture was heated for 5 min at 99 °C. The treated mixture (20 μL) was loaded onto 10% SDS gel. The gel system was applied using Schägger and Tricine SDS-PAGE [39]. The gel was run at 90 V for stacking, followed by 120 V for 40 min to separate the gel.

After protein separation, the gel was briefly rinsed with tap water. It was then fixed in fixing buffer (50% methanol, 10% acetic acid, and 100 mM ammonium acetate) for 15–30 min at room temperature. After 2–3 times washing with tap water, the gel was stained with Coomassie for 30 min. It was then washed thrice with tap water and destained in a destaining solution (10% acetic acid) until the background was clear.

#### 2.6.2. Sample Transfer and Blotting

For Western blotting, the gel was cast after protein separation without staining with Coomassie dye in an electrophoretic transfer cell (Mini Trans-Blot^®^ Cell, Bio-Rad Laboratories, Hercules, CA, USA). This procedure was performed following the manufacturer’s instructions. The sample was transferred to nitrocellulose blotting paper (Mini Trans-Blot^®^ Cell, Bio-Rad Laboratories, Hercules, CA, USA) in Towbin buffer [40] by applying 40 mA overnight at 4 °C.

The following day, the free surface on the blotting paper was blocked by incubation with 5% bovine serum albumin (BSA) in PBS for 1 h at room temperature with agitation. It was then washed once with washing buffer and incubated with serum diluted in blocking buffer (1:2000) for 1 h at 4 °C. After washing with washing buffer, the blotting paper was incubated with a diluted secondary antibody (mouse monoclonal anti-rabbit IgG (γ-chain specific)-peroxidase) (1:5000 Abcam) for 1 h at 4 °C. The sections were then washed three times with PBS and incubated with 3,3′-diaminobenzidine (DAB) peroxidase substrate solution for 5 min at room temperature with agitation. The reaction was stopped using 2N sulfuric acid. Finally, the blot paper was dried and photographed.

### 2.7. Protein Structure Comparison

The Protean 3D^®^ software of the DNAStar (Version 17.4.3. DNASTAR, Inc., Madison, WI, USA.) was used to align and compare the protein structures cited in the results section. The structures were aligned using an automated algorithm applied to the entire structures and the RMSD values were calculated with the software’s routines.

## 3. Results

### 3.1. Epitope Fingerprinting (In Vitro)

Data from selection experiments using a random peptide library with five rabbit samples immunized with hazelnut powder were matched to known allergenic hazelnut proteins. Epitopes were identified using a previously described statistical peptide phage-display method [33]. Seventy-five potential epitopes were identified using rabbit anti-hazelnut polyclonal antisera. Overall, most epitopes were recognized by antibodies from at least two rabbits. Seventeen epitopes were detected using antibodies from both rabbits. Antibodies from rabbit sera recognized motifs for epitopes of 11 allergenic hazelnut proteins (Table 1). Comparing the results from different sera allowed for the selection of peptides with a minimal size recognized by different sera.

### 3.2. Results from Array Measurements

Hazelnut peptide epitopes were synthesized and spotted as microarrays on glass slides [33]. Sera from three immunized rabbits were used for the analysis (Table 1). Rabbit IgGs bound to several epitopes that were previously identified in human serum samples. In the array experiments, at least 50% of these were found in all three rabbits, and 80% were found in at least one. Seven of these peptides showed a high signal intensity (Table 1). All peptides recognized by the rabbit serum samples were identified as IgE and/or IgG in human samples.

### 3.3. Changing the Thermal Processing to Roasting

The hazelnuts were roasted under different conditions to simulate low-temperature–long-time (LTLT) and high-temperature–short-time (HTST) processes. Two trials involved moderately long roasting times (30 and 50 min) at 142 °C, while the other two involved fast roasting (8 and 10 min) at 200 °C.

Table 2 shows whether heat treatment of a hazelnut allergen resulted in a change in the protein (heat labile) or not (heat stable).

**Figure 1:**
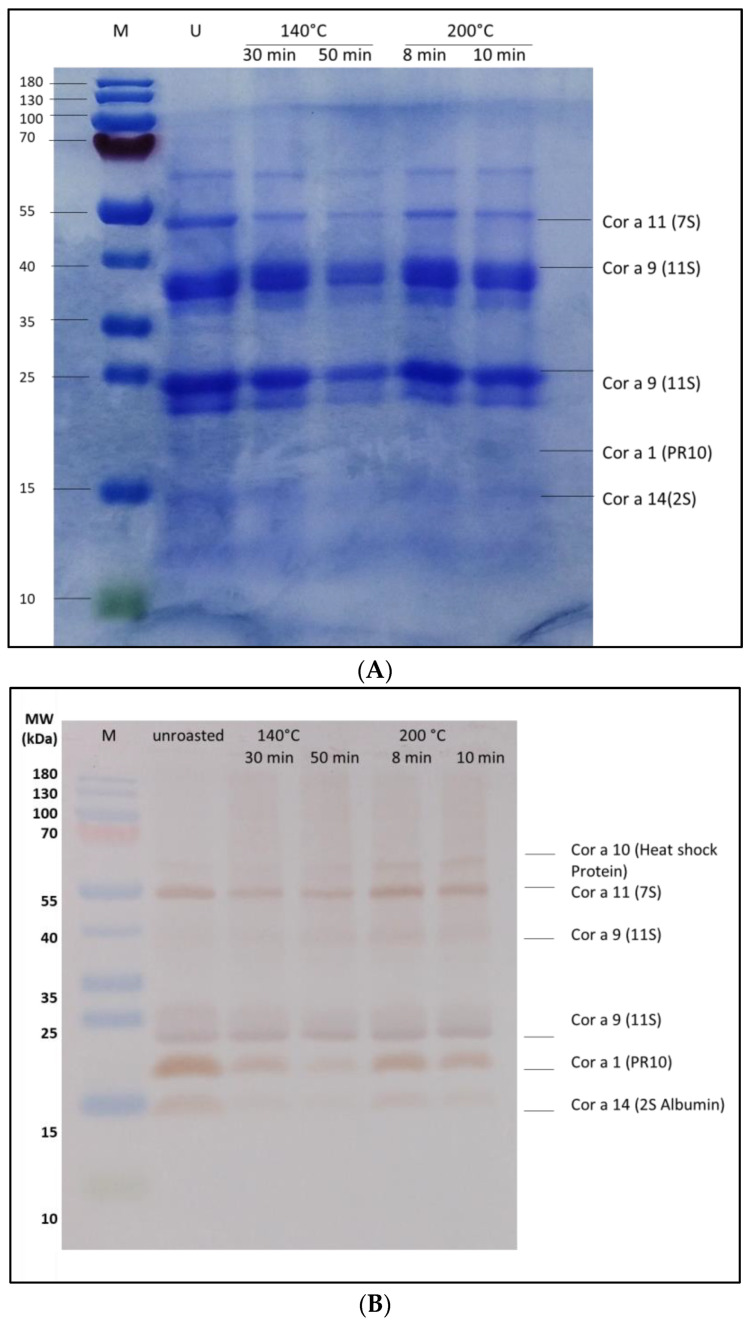

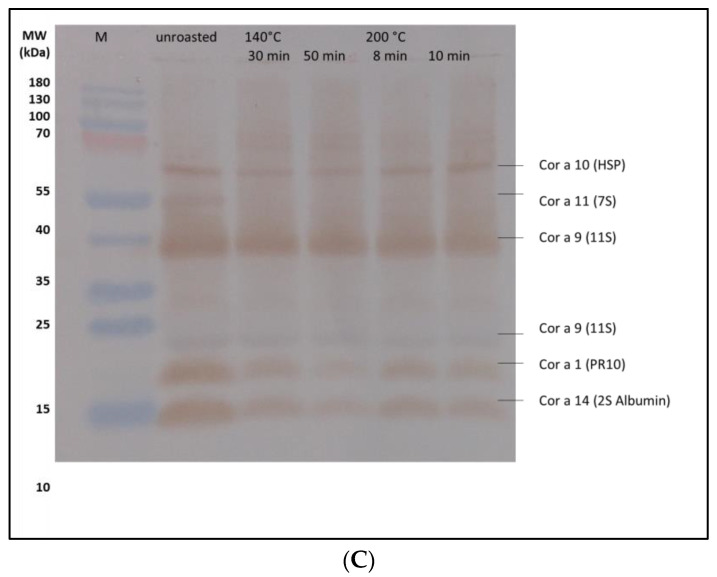
Extracts from hazelnut roasted under different conditions: Coomassie-stained SDS-PAGE (**A**) and Western blot with sera from two rabbits (**B**,**C**). (**B**) serum from rabbit 1 after immunization (diluted 1:2000); (**C**) serum from rabbit 2 after immunization (diluted 1:2000).

Coomassie-stained SDS-PAGE (Figure 1A) showed no significant alterations in protein patterns after different treatments. All major hazelnut proteins could still be found in different amounts in the soluble form of the roasted hazelnuts.

Rabbit IgG binding to extracts from roasted ground hazelnuts was further investigated using Western blotting. A band at 22 kDa was observed in all hazelnut extracts tested with the sera before immunization from both rabbits as well as when no serum was introduced into the test (see Supplementary Materials). This indicates that the secondary antibody may also bind to a hazelnut protein because the intensity of this protein band decreases upon heat treatment. Before immunization, no further bands were observed, indicating no binding to the hazelnut protein. Rabbit IgG showed strong binding to hazelnut proteins with molecular weights of 65 and 55 kDa (Cor a 11) and 22 kDa (Cor a 9) (Figure 1B,C). Faint bands were visible at 17 and 15 kDa (Cor a 1, Cor a 2, and/or Cor a 12). IgG binding to the 40 kDa (Cor a 9) hazelnut protein can be observed by immunoblotting with the serum from rabbit 2. Roasting at 140 °C for 50 min slightly decreased the amount of protein detected in the Western blot for rabbit 1, which may be due to the denaturation of epitopes or entire proteins.

### 3.4. Cross-Reactivity with Different Plant Extracts

**Figure 2:**
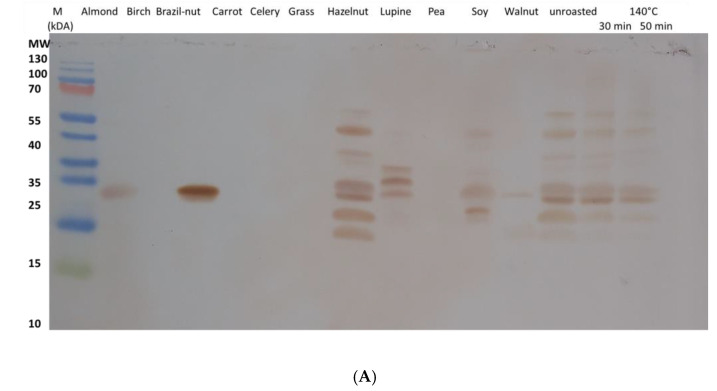

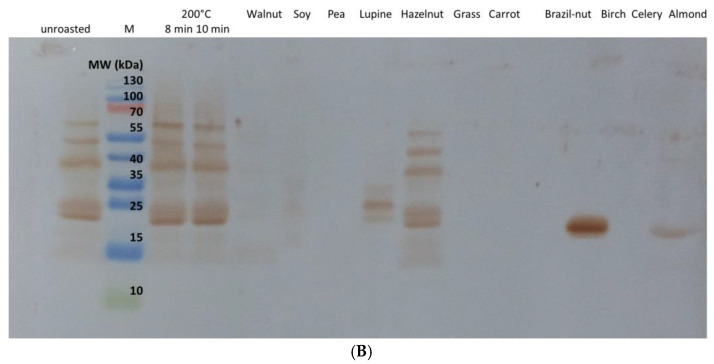
Western blots with plant extracts used in skin-prick tests of the FoodAllergen project with two rabbit sera after immunization. (**A**) Serum from rabbit 1; (**B**) serum from rabbit 2.

Western blotting of different plant extracts used in the skin-prick test was performed to study cross-reactivity with other plant proteins. Antibodies from both rabbit sera recognized the same proteins but with different signal intensities, and both rabbit sera showed binding not only to several hazelnut proteins but also to one Brazil nut and almond 22 kDa protein (11S seed storage globulin). Rabbit 1 serum strongly recognized the three lupin proteins, whereas rabbit 2 serum strongly recognized a distinct lupin protein of approximately 30 kDa. Moreover, rabbit 1 serum antibodies bound to several soy proteins, and a slight 22 kDa (walnut protein) band was observed. Rabbit 2 serum weakly recognizes these proteins (Figure 2).

**Figure 3:**
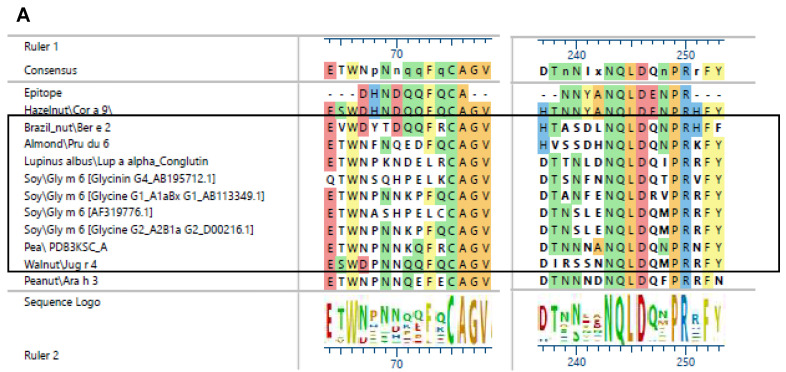

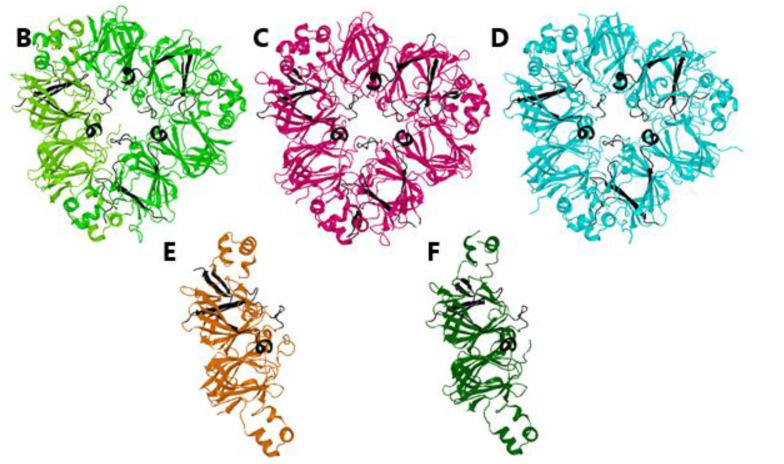
Alignment (**A**) of epitope 63-DHNDQQFQCA and 183-NNYANQLDENPR of the Cor a 9 protein with different plant species. Species detected by rabbit antibodies are marked in boxes. Structure of 11S globulins of different species soybean Gly m 6 2D5H ((**B**), green [48]), almond 3EHK ((**C**), pink [49]), pea 3KSC ((**D**), light blue [48]), peanut Ara h 3 3C3V ((**E**), orange [50]), and Brazil nut 6B4S ((**F**), dark green [51]). Identified epitopes are colored in black.

The cross-reactivity observed in the Western blot was verified by the alignment of the Cor a 9 protein (band 22 kDa). Epitope 63-DHNDQQFQCA and 183-NNYANQLDENPR shared high sequence similarities among the different plant species (Figure 3A). The structural similarities between the proteins of different plant species are shown in Figure 3B–F and their RMDS values in Table 3.

## 4. Discussion

Heat treatment or other methods to reduce the allergenicity of food by modifying allergens cannot be measured or quantified using uncharacterized polyclonal antibody mixtures. A large number of individual mAbs cannot cover the variety of individual immune responses. Basophile activation tests require patient material and provide only patient-specific information [52,53,54]. In a recent study, polyclonal rabbit serum was used for the characterization of the immune response to processed pea proteins [55]. Here, we compared the immune responses of allergic patients with those of rabbits at the epitope level. The similarity of the epitopes could be used to develop more reliable methods, perhaps even standards, to better understand the beneficial effects of food processing on allergenicity reduction.

Our data highlight the advantages of short, highly specific peptide epitopes for detailed analysis of immune responses. This enabled the characterization and comparison of antibodies against hazelnut proteins in humans and immunized animals. Several epitopes in different hazelnut allergens can be identified using rabbit anti-hazelnut polyclonal antisera. Overall, the microarray with peptides based on epitopes identified using human sera revealed that the immune system of vaccinated rabbits recognizes the same epitopes as antibodies present in the serum of individuals. Immunized rabbits have been used several times to detect the immunogenicity of food allergies [17,18,19]. Those studies showed that epitopes recognized by antibodies from human serum were also recognized by antibodies from rabbit serum. The data presented herein demonstrated that rabbit serum can be used as a surrogate for human serum. Rabbit serum offers several advantages. The B-cell response is better controlled in humans, where cross-reactivity with other plant proteins may obscure the results. It is known what rabbits have been fed on and the resulting potential food-induced immune responses. Proteins from other species detected by antibodies resulting from immunization with a given antigen were most likely due to cross-reactivity (Figure 2). The animals were unintentionally exposed to antigens.

In this study, we investigated the influence of thermal treatment on the in vitro allergenic reactivity of hazelnut proteins at the epitope level. Roasting at temperatures above 180 °C significantly changes the secondary structure. The α-helix content increases with increasing temperature, whereas the β-sheet content decreases [56]. Different heat treatment procedures result in different protein degradation products. The results of this study are in agreement with previous findings that thermal treatment reduces the IgE immunoreactivity of hazelnut proteins [13,57]. Reduced signal intensity for Cor a 1 (PR10) and Cor a 14 (2S albumin) after roasting was reported by Pastorello et al. and Lopez et al. [41,44]. Compared to Lamberti et al., discrepancies were observed for Cor a 11 (7S globulin) and Cor a 14. Lamberti et al. showed that the immune reactivity of Cor a 11 and Cor a 14 was stable after roasting at 140 °C but was reduced after roasting at 170 °C [43]. Denaturation of 2S albumins was observed at temperatures above 120 °C [58]. However, the epitopes in 2S albumins are highly resistant to severe heat treatment [59]. The immunogenicity of other 2S albumins could also be increased [60]. 7S globulin forms aggregates after heating [61], which might lead to an increase in immunoreactivity. The application of different batches of polyclonal sera characterized for recognizing different epitopes, such as R1 and R2 sera in this study, can help monitor different epitopes in the analysis of specific proteins and individual heat-labile epitopes, for example, Cor a 14.

The observed cross-reactivity in the Western blot could be well explained by the sequence alignment (Figure 3) and the highly conserved structure (Figure 3B,C). Surprisingly, denatured pea protein was not detected by Western blotting, although the structure and sequence of pea 7S globulin are very similar to those of hazelnut 7S globulin. Minor changes in the pea protein sequence are sufficient for rabbit antibodies to not detect the pea protein in the Western blot.

## 5. Conclusions

The allergenicity of food is usually assessed by measuring the total amount of allergenic proteins using monoclonal or polyclonal antibodies. Changes in immunogenicity are regarded as an increase or decrease in the allergenicity of a protein upon treatment. However, allergenic proteins contain multiple epitopes. Variations in individual patients’ antibodies can be of individual relevance to the allergenicity of a protein, even when not taking into account cross-reactive antibodies or remaining protein fragments. Overall, the reproducibility of the experiments depends on the antibodies used. This explains the different published results in Table 1. Using polyclonal antibodies that are well characterized at the epitope level could help establish reliable methods. Each of the sera used in this study would lead to different outcomes on the allergenicity of foods after treatments. In this work, we show that a combination of different sera characterized by peptide epitopes (Table 2) should help to compare and standardize immune diagnostics. We show that this is even possible for complex protein preparations. Being able to predict potential cross-reactivity, these analytes could even be mixtures from multiple allergen sources (Figure 2).

## Figures and Tables

**Table 1:** Overview of the recognition patterns of different hazelnut proteins and identified epitopes using rabbit serum (R1 and R2), Western blotting (WB), and peptide phage display with next-generation sequencing (NGS). Peptides based on the epitopes were also tested using sera from individuals sensitized with hazelnuts and/or peanuts. Numbers before “/” mean positive-tested and total numbers of tested patients. The peptide sequences are not listed in the table. Unroasted (UR) samples are shown for Western blot. See supplement for signal intensities and peptide codes.

		WB		NGS			Patient		
Protein	Mass (Da)	R1 (UR)	R2 (UR)	R1	R2	Epitope-Rabbits	IgE	IgG	IgE and IgG
Cor a 1	17.5	x	x	x	x	3-vfnYEVETps			
				x	x	32-pkVAPQa	39/230	62/235	27/229
				x	x	59-fgEGSRyky			
				x	x	94-dklEKVCSelk			
				x	x	124-kgdHEINaee	111/333	110/340	63/331
				x		9-eTPSVIsa			
				x		67-yvkERVDe			
				x		10-etPSVISa			
				x		143-llraVETYll	8/230	30/235	3/229
				x		141-aEKLLRav	43/329	39/336	13/327
						13-VIPPARLF	32/333	61/340	16/331
						38-aiTSVenvgg	25/285	54/294	12/284
Cor a 2	14.2	x	x	x	x	30-hdGSVWaqsssf	114/333	117/340	69/331
				x	x	11-lmcdiDGQGQq			
				x	x	1-swqaYVDEhl			
				x	x	104-giyeEPVTPgqc			
				x		25-aSAIVGhd			
						45-kPEEItg			
						51-IKDFDEPGSLA	72/329	104/340	48/331
						58-epGHLAPtg			
Cor a 6	34.2			x	x	19-fivEASLkag			
				x	x	238-lekiHLTEEKl			
				x	x	298-tvEEYLqqf			
				x		248-ilKDIQEspi			
				x		281-eESFGVe			
				x		290-qLYPDVky			
					x	39-tVSDPvk			
				x		63-dlyDHGSlv			
				x		119-drVHAVEp			
				x		131-atKVEIRrk			
				x		141-EAEGIPYTY			
				x		202-vDDPRtl			
				x		72-lvkaIKHVDVv			
Cor a 8	9			x	x	73-nCLKDtak			
					x	24-slTCPQik	80/333	109/340	59/284
				x		33-nlTPCVLy			
				x		104-kispsTNCNnv	46/329	75/336	22/327
						5-kLVCAvllc	7/329	8/173	6/169
						49-PSCCKGVRA	68/333	74/340	36/331
Cor a 9	58.8			x	x	63-dhnDQQFqc			
basic subunits	22	x	x	x	x	289-rqewERQErqere			
acidic subunit	40	x	x	x	x	106-itgVLFPgcp	75/285	108/294	60/284
				x	x	119-EDPQQQs			
				x	x	160-agVAHWCyndg			
					x	59-ieSWDHn			
				x		262-rlQSNQdk	66/333	90/340	49/331
					x	302-seQERERqrrQGGRg	39/167	62/340	25/331
				x		131-qGQGQSq			
				x		141-QDRHQk	25/285	34/294	10/284
				x		185-YANQLDe			
					x	201-npddEHQrQGQQQFgqr			
					x	237-nVFSGfd_ef			
				x		277-egRLQVVRPer			
				x		340-r_diYTEQVgr			
				x		411-VFDDelr	71/171	90/336	35/327
						354-vnsnTLPVLrwlql	23/171	35/173	13/169
Cor a 10	73.5	x		x	x	16-ilFGCLfai			
				x	x	55-ngHVELia			
				x	x	111-edKEVQkd			
				x	x	236-tfDVSILTIDNgvf			
				x	x	296-rREAEra			
				x	x	305-iSSQHQVRvries			
				x	x	361-nQIDEIvLVGGs			
				x	x	382-iKDYFdgk			
				x	x	460-ftTYQDQqtv			
				x	x	468-tvSIQVFege			
					x	70-sWVGFtdg			
				x		205-iiNEPTaa			
						257-dtHLGGedf			
				x		322-GVDFSepltr			
				x		397-pdEAVAYgaa			
				x		433-lGIETvgg			
						615-dDNQSAeke			
						629-lkEVEAVCnp			
Cor a 11	48	x	x	x	x	421-fkNQDQAff			
				x		46-gnSSEESyg	71/333	87/336	37/327
				x		74-kteeGRVQVLENftk			
				x		139-kreSFNVEhgd			
				x		192-gGEDPeSfY			
				x		338-ssSGSYQki			
						14-kcRDERQf	50/333	33/340	17/331
						40-ERQQEE			
						56-eqeeNPYVF	47/333	84/340	31/331
Cor a 12	17	x	x	x		149-iqSRAQegr			
						7-QLQVHPQRGHG	94/333	109/340	64/331
						121-EMKDRAEQFGQQHV	64/333	73/340	27/331
Cor a 13	14–16				x	14-qpRSHQvvka			
Cor a 14	12	x	x	x	x	30-vdvDEDivn			
				x	x	45-eSCREQAQRQqnl	19/285	52/294	9/284
				x	x	55-qnlNQCQry			
						134-rlspQRCEirsARf	91/333	86/340	13/331
						38-NQQGRR	49/333	69/336	27/327

**Table 2:** Hazelnut allergens and their behavior to heat treatment are described in the literature and Western blot (R1 = rabbit 1 and R2 = rabbit2).

Protein	Characteristic	Protein Class	Western Blot
Cor a 1	Heat-labile [41]		Heat-labile
Cor a 10	Heat-stable [42]		Heat-stable
Cor a 11	Heat-stable [43]; heat-labile [44]		Heat-stable (R1); heat-labile (R2)
Cor a 12	Heat-stable	Oleosin [45]	
Cor a 13	Heat-stable	Oleosin [45]	
Cor a 14	Heat-stable [43]		Heat-stable (R2); heat-labile (R1)
Cor a 15	Heat-stable	Oleosin [45]	
Cor a 2	Heat-stable [46]; heat-labile [47]		
Cor a 6	n.d.	Isoflavone Reductase	
Cor a 8	Heat-stable [41]; heat-labile [43,44]		
Cor a 9	Heat-stable [43]; heat-labile [44]		Heat-stable (R1, R2)

**Table 3:** RMSD values of structure alignments.

	2D5H	3C3V	3EHK	3KSC	6B4S
2D5H		1386	1309	1397	1283
3C3V	1386		1623	1627	
3EHK	1309	1623			
3KSC	1397	1627			
6B4S	1283				

## Data Availability

The original contributions presented in the study are included in the article and Supplementary Materials, further inquiries can be directed to the corresponding author.

## Supplementary Materials

The following supporting information can be downloaded at https://www.mdpi.com/article/10.3390/foods13233932/s1: Table S1 showing the measured intensities with a peptide microarray covering the epitopes (Table S1) for different rabbit sera; Table S2 shows a complete version of Table 2. Figure S1 shows the extracts from hazelnut roasted under different conditions at timepoint T0.
