# Supplementary material for: Heat Treatment of Hazelnut Allergens Monitored by Polyclonal Sera and Epitope Fingerprinting
Source: Foods. 2024 Dec 5;13(23):3932. doi: 10.3390/foods13233932 (PMC11640838; doi:10.3390/foods13233932)
Supplement: Supplementary file 1 [file foods-13-03932-s001.zip › 241115_Suppl_Table-S2.pdf]

		WB		NGS				Patient		
**Protein**	**Mass (Da)**	**R1 (UR)**	**R2 (UR)**	**R1**	R2	Epitope-Rabbits	Peptide	IgE	IgG	IgE&IgG
Cor a 1	17,5	x	x	x	x	3-vfnYEVETps				
				x	x	32-pkVAPQa	S7*	39/230	62/235	27/229
				x	x	59-fgEGSRyky				
				x	x	94-dklEKVCSelk				
				x	x	124-kgdHEINaee	FX15	111/333	110/340	63/331
				x		9-eTPSVIsa				
				x		67-yvkERVDe				
				x		10-etPSVISa				
				x		143-llraVETYll	II5	8/230	30/235	3/229
				x		141-aEKLLRav	HN11/HN12	43/329	39/336	13/327
						13-VIPPARLF	FX12	32/333	61/340	16/331
						38- aiTSVenvgg	FJ19/FJ20/S6*	25/285	54/294	12/284
Cor a 2	14,2	x	x	x	x	30-hdGSVWaqsssf	HN19/HN20*	114/333	117/340	69/331
				x	x	11-lmcdiDGQGQq				
				x	x	1-swqaYVDEhl				
				x	x	104-giyeEPVTPgqc				
				x		25-aSAIVGhd				
						45-kPEEItg				
						51-IKDFDEPGSLA	GL7/GL8	72/329	104/340	48/331
						58-epGHLAPtg				
Cor a 6	34,2			x	x	19-fivEASLkag				
				x	x	238-lekiHLTEEKl				
				x	x	298-tvEEYLqqf				
				x		248-ilKDIQEspi				
				x		281-eESFGVe				
				x		290-qLYPDVky				
					x	39-tVSDPvk				
				x		63-dlyDHGSlv				
				x		119-drVHAVEp				
				x		131- atKVEIRrk				
				x		141-EAEGIPYTY				
				x		202-vDDPRtl				
				x		72-lvkaIKHVDVv				
Cor a 8	9			x	x	73-nCLKDtak				
					x	24-slTCPQik	HU15*	80/333	109/340	59/284
				x		33-nlTPCVLy				
				x		104-kispsTNCNnv	FX18	46/329	75/336	22/327
						5-kLVCAvllc	FZ1	7/329	8/173	6/169
						49-PSCCKGVRA	FX16	68/333	74/340	36/331

Protein		WB		NGS				Patient		
	Mass (DA)	R1 (UR)	R2 (UR)	R1	R2	Epitope-Rabbits	Peptide	IgE	IgG	IgE&IgG
Cor a 9	58,8			x	x	63-dhnDQQFqc				
basic subunits	22	x	x	x	x	289-rqewERQErqere				
acidic subunit	40	x	x	x	x	106-itgVLFPgcp	DL3*	75/285	108/294	60/284
				x	x	119-EDPQQQs				
				x	x	160-agVAHWCyndg				
					x	59-ieSWDHn				
				x		262-rlQSNQdk	FX20*	66/333	90/340	49/331
					x	302-seQERERqrrQGGRg	FX21/FX22	39/167	62/340	25/331
				x		131-qGQGQSq				
				x		141-QDRHQk	D022	25/285	34/294	10/284
				x		185-YANQLDe				
					x	201-npddEHQrQGQQQFgqr				
					x	237-nVFSGfd_ef				
				x		277-egRLQVVRPer				
				x		340-r_diYTEQVgr				
				x		411-VFDDelr	GL1	71/171	90/336	35/327
						354-vnsnTLPVLrwlql	FX19	23/171	35/173	13/169
Cor a 10	73,5	x		x	x	16-ilFGCLfai				
				x	x	55-ngHVELia				
				x	x	111-edKEVQkd				
				x	x	236-tfDVSILTIDNgvf				
				x	x	296-rREAEra				
				x	x	305-iSSQHQVRvries				
				x	x	361-nQIDEIvLVGGs				
				x	x	382-iKDYFdgk				
				x	x	460-ftTYQDQqtv				
				x	x	468-tvSIQVFege				
					x	70-sWVGFtdg				
				x		205-iiNEPTaa				
				x		322-GVDFSepltr				
				x		397-pdEAVAYgaa				

				x		433-lGIETvgg				
						615-dDNQSAeke				
		WB		NGS				Patient		
Protein	Mass (Da)	R1 (UR)	R2 (UR)	R1	R2	Epitope-Rabbits	Peptide	IgE	IgG	IgE&IgG
Cor a 10						257-dtHLGGedf				
						629-lkEVEAVCnp				
Cor a 11	48	x	x	x	x	421-fkNQDQAff				
				x		46-gnSSEESyg	FY1	71/333	87/336	37/327
				x		74-kteeGRVQVLENftk				
				x		139-kreSFNVEhgd				
				x		192-gGEDPeSfY				
				x		338-ssSGSYQki				
						14-kcRDERQf	FX23	50/333	33/340	17/331
						40-ERQQEE				
						56-eqeeNPYVF	FX24*	47/333	84/340	31/331
Cor a 12	17	x	x	x		149-iqSRAQegr				
						7-QLQVHPQRGHG	HN15/HN16	94/333	109/340	64/331
						121-EMKDRAEQFGQQHV	HN17/HN18	64/333	73/340	27/331
Cor a 13	14-16				x	14-qpRSHQvvka				
Cor a 14	12	x	x	x	x	30-vdvDEDivn				
				x	x	45-eSCREQAQRQqnl	FH19/FH20	19/285	52/294	9/284
				x	x	55-qnlNQCQry				
						134-rlspQRCEirsARf	HN13/HN14	91/333	86/340	13/331
						38-NQQGRR	GL9/GL10/GL11	49/333	69/336	27/327
