# Supplementary figures and images for: Heat Treatment of Hazelnut Allergens Monitored by Polyclonal Sera and Epitope Fingerprinting
Source: Foods. 2024 Dec 5;13(23):3932. doi: 10.3390/foods13233932 (PMC11640838; doi:10.3390/foods13233932)

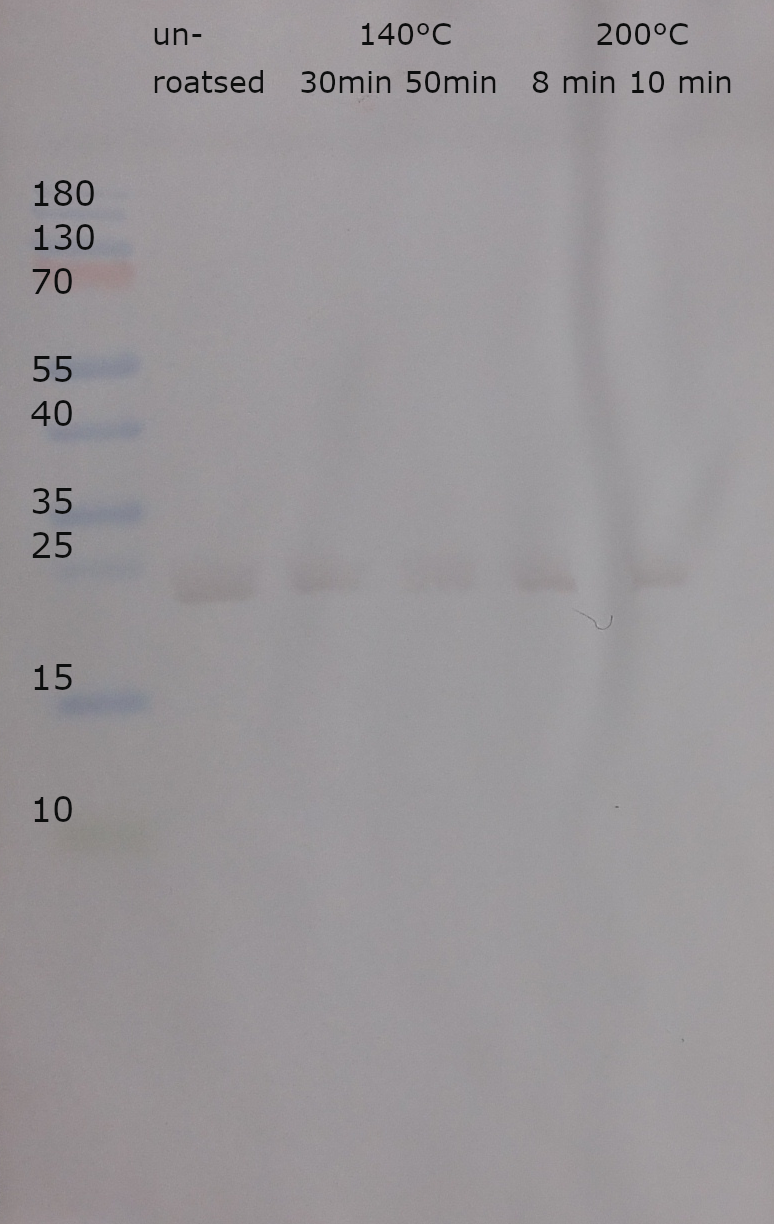

Supplement: Supplementary file 1 [file foods-13-03932-s001.zip › thermal_processing_T0-S3.png]
